# (*S*)-(–)-Methyl 2-(*p*-tolyl­sulfon­yloxy)­propanoate

**DOI:** 10.1107/S1600536808010064

**Published:** 2008-04-18

**Authors:** Wei Chen, Shan Liu, Qing-Yan Chu, Wei Luo, Hong-Jun Zhu

**Affiliations:** a Department of Applied Chemistry, College of Science, Nanjing University of Technology, Nanjing 210009, People’s Republic of China

## Abstract

In the title compound, C_11_H_14_O_5_S, there is an intra­molecular C—H⋯O hydrogen bond, for which the C—C—S—O torsion angle involving the acceptor and donor atoms is 2.4 (4)°. The dihedral angle between the benzene ring and the methoxy­carbonyl plane is 52.7 (4)°. In the crystal structure, mol­ecules are linked *via* inter­molecular C—H⋯O hydrogen bonds, forming a mol­ecular chain along the *b* axis.

## Related literature

For related literature, see: Allen *et al.* (1987 ▶); Chan *et al.* (1975 ▶); Talbert *et al.* (1974 ▶).
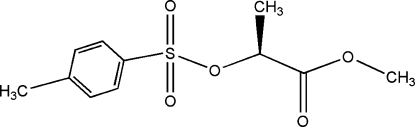


## Experimental

### 

#### Crystal data

C_11_H_14_O_5_S
*M* *_r_* = 258.28
Orthorhombic, 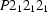

*a* = 7.4890 (15) Å
*b* = 10.150 (2) Å
*c* = 17.362 (4) Å
*V* = 1319.7 (5) Å^3^
*Z* = 4
Mo *K*α radiation
μ = 0.25 mm^−1^
*T* = 298 (2) K
0.40 × 0.20 × 0.20 mm

#### Data collection

Enraf–Nonius CAD-4 diffractometer
Absorption correction: ψ scan (North *et al.*, 1968 ▶) *T*_min_ = 0.906, *T*_max_ = 0.951
2933 measured reflections
2581 independent reflections
1703 reflections with *I* > 2σ(*I*)
*R*_int_ = 0.063
3 standard reflections every 200 reflections intensity decay: none

#### Refinement

*R*[*F* ^2^ > 2σ(*F* ^2^)] = 0.063
*wR*(*F* ^2^) = 0.154
*S* = 1.01
2581 reflections
154 parameters
H-atom parameters constrained
Δρ_max_ = 0.31 e Å^−3^
Δρ_min_ = −0.29 e Å^−3^
Absolute structure: Flack (1983 ▶), 1073 Friedel pairs
Flack parameter: 0.20 (16)

### 

Data collection: *CAD-4 Software* (Enraf–Nonius, 1985 ▶); cell refinement: *CAD-4 Software*; data reduction: *XCAD4* (Harms & Wocadlo, 1995 ▶); program(s) used to solve structure: *SHELXS97* (Sheldrick, 2008 ▶); program(s) used to refine structure: *SHELXL97* (Sheldrick, 2008 ▶); molecular graphics: *SHELXTL* (Sheldrick, 2008 ▶); software used to prepare material for publication: *SHELXL97*.

## Supplementary Material

Crystal structure: contains datablocks I, global. DOI: 10.1107/S1600536808010064/is2285sup1.cif

Structure factors: contains datablocks I. DOI: 10.1107/S1600536808010064/is2285Isup2.hkl

Additional supplementary materials:  crystallographic information; 3D view; checkCIF report

## Figures and Tables

**Table 1:** Hydrogen-bond geometry (Å, °)

*D*—H⋯*A*	*D*—H	H⋯*A*	*D*⋯*A*	*D*—H⋯*A*
C4—H4*A*⋯O1	0.93	2.53	2.910 (6)	104
C4—H4*A*⋯O3^i^	0.93	2.52	3.297 (5)	141
C6—H6*A*⋯O1^ii^	0.93	2.55	3.478 (6)	172

Symmetry codes: (i) 
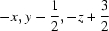
; (ii) 
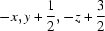
.

**Fig. 1.**
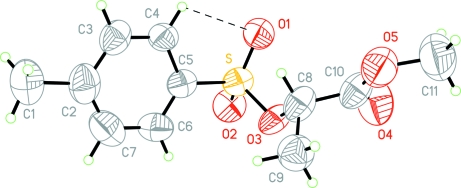
A drawing of the title molecular structure, with the atom-numbering scheme. Displacement ellipsoids are drawn at the 50% probability level. The hydrogen bond is shown by dashed line.

**Fig. 2.**
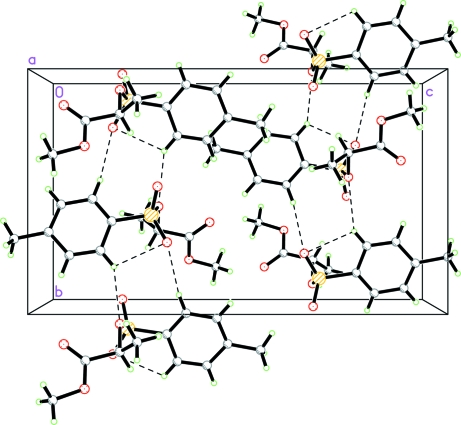
A packing diagram of the title compound, viewed along the a axis. Hydrogen bonds are shown as dashed lines.

## supplementary crystallographic information

### Comment

(*S*)-(-)-methyl 2-(*p*-toluenesulfonyloxy)propanoate is an important fine chemical, which can be used for many fields such as chiral pesticide, organometallic chemistry, *etc*. (Talbert *et al.*, 1974). The bond lengths and angles of the title compound are within normal ranges (Allen *et al.*, 1987). In the crystal structure, molecules are linked *via* intermolecular C—H···O hydrogen bonds, which with intramolecular C—H···O hydrogen bonds seem to be effective in the stabilization of the crystal. As can be seen from the packing diagram (Fig. 2), the molecules are stacked along the *a* axis.

### Experimental

The title compound was prepared according to the literature method (Chan *et al.*, 1975). The crystals were obtained by dissolving the title compound (500 mg, 2 mmol) in ethyl acetate (20 ml) and evaporating the solvent slowly at room temperature for about 7 d.

### Refinement

H atoms were positioned geometrically (C—H = 0.93–0.98 Å) and constrained to ride on their parent atoms, with *U*_iso_(H) = 1.2*U*_eq_(C) or 1.5*U*_eq_(methyl C).

### Crystal data

**Table:**

C_11_H_14_O_5_S	*F*_000_ = 544
*M**_r_* = 258.28	*D*_x_ = 1.300 Mg m^−^^3^
Orthorhombic, *P*2_1_2_1_2_1_	Mo *K*α radiation λ = 0.71073 Å
Hall symbol: P 2ac 2ab	Cell parameters from 25 reflections
*a* = 7.4890 (15) Å	θ = 9–14º
*b* = 10.150 (2) Å	µ = 0.25 mm^−^^1^
*c* = 17.362 (4) Å	*T* = 298 (2) K
*V* = 1319.7 (5) Å^3^	Block, colorless
*Z* = 4	0.40 × 0.20 × 0.20 mm

### Data collection

**Table:**

Enraf–Nonius CAD-4 diffractometer	*R*_int_ = 0.063
Radiation source: fine-focus sealed tube	θ_max_ = 26.0º
Monochromator: graphite	θ_min_ = 2.3º
*T* = 298(2) K	*h* = −9→9
ω/2θ scans	*k* = 0→12
Absorption correction: ψ scan(North *et al.*, 1968)	*l* = 0→21
*T*_min_ = 0.906, *T*_max_ = 0.951	3 standard reflections
2933 measured reflections	every 200 reflections
2581 independent reflections	intensity decay: none
1703 reflections with *I* > 2σ(*I*)	

### Refinement

**Table:**

Refinement on *F*^2^	Hydrogen site location: inferred from neighbouring sites
Least-squares matrix: full	H-atom parameters constrained
*R*[*F*^2^ > 2σ(*F*^2^)] = 0.063	*w* = 1/[σ^2^(*F*_o_^2^) + (0.06*P*)^2^ + *P*] where *P* = (*F*_o_^2^ + 2*F*_c_^2^)/3
*wR*(*F*^2^) = 0.154	(Δ/σ)_max_ < 0.001
*S* = 1.01	Δρ_max_ = 0.31 e Å^−^^3^
2581 reflections	Δρ_min_ = −0.29 e Å^−^^3^
154 parameters	Extinction correction: none
Primary atom site location: structure-invariant direct methods	Absolute structure: Flack (1983), 1073 Friedel pairs
Secondary atom site location: difference Fourier map	Flack parameter: 0.20 (16)

### Special details

**Table:**

Geometry. All e.s.d.'s (except the e.s.d. in the dihedral angle between two l.s. planes) are estimated using the full covariance matrix. The cell e.s.d.'s are taken into account individually in the estimation of e.s.d.'s in distances, angles and torsion angles; correlations between e.s.d.'s in cell parameters are only used when they are defined by crystal symmetry. An approximate (isotropic) treatment of cell e.s.d.'s is used for estimating e.s.d.'s involving l.s. planes.
Refinement. Refinement of *F*^2^ against ALL reflections. The weighted *R*-factor *wR* and goodness of fit *S* are based on *F*^2^, conventional *R*-factors *R* are based on *F*, with *F* set to zero for negative *F*^2^. The threshold expression of *F*^2^ > σ(*F*^2^) is used only for calculating *R*-factors(gt) *etc*. and is not relevant to the choice of reflections for refinement. *R*-factors based on *F*^2^ are statistically about twice as large as those based on *F*, and *R*- factors based on ALL data will be even larger.

### Fractional atomic coordinates and isotropic or equivalent isotropic displacement parameters (Å^2^)

**Table:**

	*x*	*y*	*z*	*U*_iso_*/*U*_eq_	
S	0.06641 (15)	0.89958 (12)	0.71844 (7)	0.0628 (3)	
O1	0.1083 (4)	0.7829 (3)	0.67754 (18)	0.0765 (10)	
O2	0.1557 (5)	1.0219 (3)	0.7003 (2)	0.0830 (10)	
O3	−0.1372 (4)	0.9339 (3)	0.70538 (18)	0.0689 (9)	
O4	−0.3001 (6)	0.8618 (5)	0.5745 (2)	0.1125 (16)	
O5	−0.4188 (5)	0.6878 (4)	0.6310 (2)	0.0984 (12)	
C1	0.1382 (8)	0.7979 (7)	1.0589 (3)	0.102 (2)	
H1B	0.1073	0.8750	1.0881	0.152*	
H1C	0.2598	0.7741	1.0695	0.152*	
H1D	0.0608	0.7266	1.0732	0.152*	
C2	0.1173 (7)	0.8265 (7)	0.9740 (3)	0.0853 (17)	
C3	0.1522 (6)	0.7282 (5)	0.9202 (3)	0.0723 (13)	
H3A	0.1874	0.6457	0.9377	0.087*	
C4	0.1371 (6)	0.7478 (4)	0.8434 (3)	0.0589 (11)	
H4A	0.1627	0.6807	0.8086	0.071*	
C5	0.0814 (5)	0.8730 (4)	0.8176 (3)	0.0561 (11)	
C6	0.0417 (8)	0.9679 (5)	0.8676 (3)	0.0805 (14)	
H6A	0.0034	1.0498	0.8500	0.097*	
C7	0.0576 (9)	0.9444 (6)	0.9464 (3)	0.0910 (17)	
H7A	0.0267	1.0106	0.9810	0.109*	
C8	−0.2695 (6)	0.8272 (5)	0.7102 (3)	0.0715 (13)	
H8A	−0.2174	0.7494	0.7350	0.086*	
C9	−0.4204 (7)	0.8807 (6)	0.7576 (3)	0.0969 (17)	
H9A	−0.3782	0.9008	0.8084	0.145*	
H9B	−0.5139	0.8162	0.7607	0.145*	
H9C	−0.4659	0.9593	0.7339	0.145*	
C10	−0.3253 (6)	0.7955 (6)	0.6289 (3)	0.0758 (14)	
C11	−0.5033 (10)	0.6511 (7)	0.5593 (4)	0.124 (3)	
H11A	−0.5745	0.5736	0.5671	0.186*	
H11B	−0.4134	0.6334	0.5213	0.186*	
H11C	−0.5782	0.7219	0.5420	0.186*	

### Atomic displacement parameters (Å^2^)

**Table:**

	*U*^11^	*U*^22^	*U*^33^	*U*^12^	*U*^13^	*U*^23^
S	0.0524 (6)	0.0593 (6)	0.0768 (7)	−0.0042 (6)	0.0029 (6)	0.0051 (6)
O1	0.077 (2)	0.080 (2)	0.072 (2)	0.0056 (18)	−0.0007 (17)	−0.0045 (17)
O2	0.078 (2)	0.078 (2)	0.093 (3)	−0.0125 (18)	0.0042 (18)	0.0086 (19)
O3	0.0576 (17)	0.0589 (18)	0.090 (2)	0.0055 (14)	−0.0090 (16)	0.0238 (16)
O4	0.142 (4)	0.119 (4)	0.076 (3)	−0.042 (3)	−0.004 (2)	0.026 (3)
O5	0.098 (3)	0.081 (2)	0.116 (3)	−0.011 (2)	−0.025 (2)	0.012 (2)
C1	0.092 (4)	0.137 (6)	0.075 (4)	−0.009 (4)	−0.007 (3)	−0.003 (4)
C2	0.057 (3)	0.118 (5)	0.081 (4)	−0.014 (3)	−0.012 (3)	−0.005 (4)
C3	0.058 (3)	0.070 (3)	0.089 (4)	−0.009 (2)	−0.017 (2)	0.008 (3)
C4	0.061 (3)	0.043 (2)	0.072 (3)	−0.0061 (19)	−0.006 (2)	−0.001 (2)
C5	0.036 (2)	0.052 (3)	0.080 (3)	−0.0096 (19)	−0.002 (2)	0.001 (2)
C6	0.090 (4)	0.061 (3)	0.090 (4)	0.011 (3)	0.004 (3)	−0.005 (3)
C7	0.087 (4)	0.096 (4)	0.090 (4)	−0.012 (4)	0.007 (3)	−0.032 (3)
C8	0.053 (2)	0.076 (3)	0.085 (4)	−0.010 (2)	−0.008 (2)	0.023 (3)
C9	0.078 (3)	0.114 (4)	0.099 (4)	−0.010 (4)	0.017 (3)	0.005 (3)
C10	0.053 (3)	0.085 (4)	0.089 (4)	−0.005 (3)	−0.004 (3)	0.021 (3)
C11	0.140 (7)	0.111 (5)	0.121 (6)	−0.019 (5)	−0.036 (4)	−0.009 (4)

### Geometric parameters (Å, °)

**Table:**

S—O1	1.416 (3)	C4—C5	1.411 (6)
S—O2	1.444 (3)	C4—H4A	0.9300
S—O3	1.581 (3)	C5—C6	1.331 (6)
S—C5	1.746 (5)	C6—C7	1.395 (8)
O3—C8	1.470 (5)	C6—H6A	0.9300
O4—C10	1.174 (6)	C7—H7A	0.9300
O5—C10	1.299 (6)	C8—C9	1.500 (7)
O5—C11	1.444 (7)	C8—C10	1.507 (7)
C1—C2	1.511 (7)	C8—H8A	0.9800
C1—H1B	0.9600	C9—H9A	0.9600
C1—H1C	0.9600	C9—H9B	0.9600
C1—H1D	0.9600	C9—H9C	0.9600
C2—C7	1.364 (8)	C11—H11A	0.9600
C2—C3	1.392 (8)	C11—H11B	0.9600
C3—C4	1.353 (6)	C11—H11C	0.9600
C3—H3A	0.9300		
			
O1—S—O2	120.5 (2)	C5—C6—H6A	120.1
O1—S—O3	109.0 (2)	C7—C6—H6A	120.1
O2—S—O3	103.07 (19)	C2—C7—C6	121.5 (5)
O1—S—C5	110.5 (2)	C2—C7—H7A	119.3
O2—S—C5	108.5 (2)	C6—C7—H7A	119.3
O3—S—C5	103.75 (18)	O3—C8—C9	105.8 (4)
C8—O3—S	118.7 (3)	O3—C8—C10	106.9 (4)
C10—O5—C11	115.4 (5)	C9—C8—C10	112.5 (4)
C2—C1—H1B	109.5	O3—C8—H8A	110.5
C2—C1—H1C	109.5	C9—C8—H8A	110.5
H1B—C1—H1C	109.5	C10—C8—H8A	110.5
C2—C1—H1D	109.5	C8—C9—H9A	109.5
H1B—C1—H1D	109.5	C8—C9—H9B	109.5
H1C—C1—H1D	109.5	H9A—C9—H9B	109.5
C7—C2—C3	117.0 (5)	C8—C9—H9C	109.5
C7—C2—C1	123.0 (6)	H9A—C9—H9C	109.5
C3—C2—C1	119.9 (6)	H9B—C9—H9C	109.5
C4—C3—C2	122.8 (5)	O4—C10—O5	126.3 (6)
C4—C3—H3A	118.6	O4—C10—C8	125.9 (5)
C2—C3—H3A	118.6	O5—C10—C8	107.6 (5)
C3—C4—C5	118.0 (4)	O5—C11—H11A	109.5
C3—C4—H4A	121.0	O5—C11—H11B	109.5
C5—C4—H4A	121.0	H11A—C11—H11B	109.5
C6—C5—C4	120.8 (4)	O5—C11—H11C	109.5
C6—C5—S	121.1 (4)	H11A—C11—H11C	109.5
C4—C5—S	118.1 (3)	H11B—C11—H11C	109.5
C5—C6—C7	119.8 (5)		
			
O1—S—O3—C8	44.2 (4)	C4—C5—C6—C7	0.9 (8)
O2—S—O3—C8	173.3 (3)	S—C5—C6—C7	−179.0 (4)
C5—S—O3—C8	−73.6 (4)	C3—C2—C7—C6	−3.5 (9)
C7—C2—C3—C4	3.2 (8)	C1—C2—C7—C6	179.3 (5)
C1—C2—C3—C4	−179.6 (5)	C5—C6—C7—C2	1.6 (9)
C2—C3—C4—C5	−0.8 (7)	S—O3—C8—C9	135.0 (4)
C3—C4—C5—C6	−1.3 (6)	S—O3—C8—C10	−104.8 (4)
C3—C4—C5—S	178.7 (3)	C11—O5—C10—O4	−2.2 (9)
O1—S—C5—C6	−177.6 (4)	C11—O5—C10—C8	172.4 (5)
O2—S—C5—C6	48.2 (5)	O3—C8—C10—O4	−15.7 (7)
O3—S—C5—C6	−60.9 (4)	C9—C8—C10—O4	100.0 (7)
O1—S—C5—C4	2.4 (4)	O3—C8—C10—O5	169.7 (4)
O2—S—C5—C4	−131.7 (3)	C9—C8—C10—O5	−74.6 (6)
O3—S—C5—C4	119.2 (3)		

### Hydrogen-bond geometry (Å, °)

**Table:**

*D*—H···*A*	*D*—H	H···*A*	*D*···*A*	*D*—H···*A*
C4—H4A···O1	0.93	2.53	2.910 (6)	104
C4—H4A···O3^i^	0.93	2.52	3.297 (5)	141
C6—H6A···O1^ii^	0.93	2.55	3.478 (6)	172

Symmetry codes: (i) −*x*, *y*−1/2, −*z*+3/2; (ii) −*x*, *y*+1/2, −*z*+3/2.
